# Minimally invasive resection of a giant esophageal schwannoma

**DOI:** 10.12669/pjms.40.10.8824

**Published:** 2024-11

**Authors:** Sajida Qureshi, Sumayah Khan, Waqas Ahmad Abbasi, Muneeba Sohail

**Affiliations:** 1 Sajida Qureshi, FCPS, FRCS Meritorious Professor of Surgery, Dow Medical College, Dow University of Health Sciences, Karachi-74200, Pakistan; 2 Sumayah Khan, FCPS Fellow Upper GI Surgery, Dow Medical College, Dow University of Health Sciences, Karachi-74200, Pakistan; 3 Waqas Ahmad Abbasi, BS Biosciences, MS Biosciences Research Associate, Dow Medical College, Dow University of Health Sciences, Karachi-74200, Pakistan; 4 Muneeba Sohail, MBBS Dow Medical College, Dow University of Health Sciences, Karachi-74200, Pakistan

**Keywords:** Esophageal benign tumor, Esophageal schwannoma, Giant schwannoma, McKeown esophagectomy, Minimally invasive esophagectomy

## Abstract

Benign tumors of the esophagus are uncommon, and primary esophageal schwannoma of the esophagus is even rarer, accounting for 2% of cases. Less than 30 cases have been reported in the literature. Here we report a case of a young man with a symptomatic giant esophageal schwannoma, which was completely removed by laparoscopic three-staged esophagectomy. This is the first case of such a large lesion being removed by a minimally invasive approach. Our patient was a 22-year-old male, presented with dysphagia and dyspnea for five years. An endoscopy and CT scan suggested a giant leiomyoma. Postoperative biopsy revealed a primary esophageal schwannoma. We present our case of benign esophageal tumor measuring about 10.5 cm in greatest dimension. This is the first schwannoma resection performed with the Mckeown technique among the limited case reports in the literature. Esophageal schwannoma must be kept as a possible diagnosis in patients presenting with benign esophageal tumors. Esophagectomy is the mainstay of treatment for giant esophageal schwannomas. Minimally invasive esophagectomy can be safely performed for giant benign esophageal tumors.

## CASE PRESENTATION

A 22-year-old male from rural Sindh, Pakistan, with no prior medical history, presented with a five-year history of progressive difficulty swallowing, initially with liquids and later with solids, accompanied by undocumented weight loss. He also complained of breathing difficulties when lying down and after exertion for the past year. Also, the physical examination was unremarkable. He underwent several diagnostic investigations including barium swallow, endoscopy, and CT chest with contrast. However endoscopic ultrasound could not be done because of the equipment being out of order. Barium swallow study revealed a broad-based filling defect with extraluminal indentation suggestive of an intramural esophageal lesion compressing the esophagus and causing proximal stasis. Computed tomogram showed a heterogeneously enhancing soft tissue mass arising from the left lateral wall of the esophagus, extending from D1 till D6 vertebral, anteriorly compressing trachea and the origin of both bronchi, abutting the great vessels, measuring 10.5*7.5*6.1 cm in CC*TS*AP diameter (Fig.1A) suggestive of esophageal leiomyoma.

**Fig.1:**
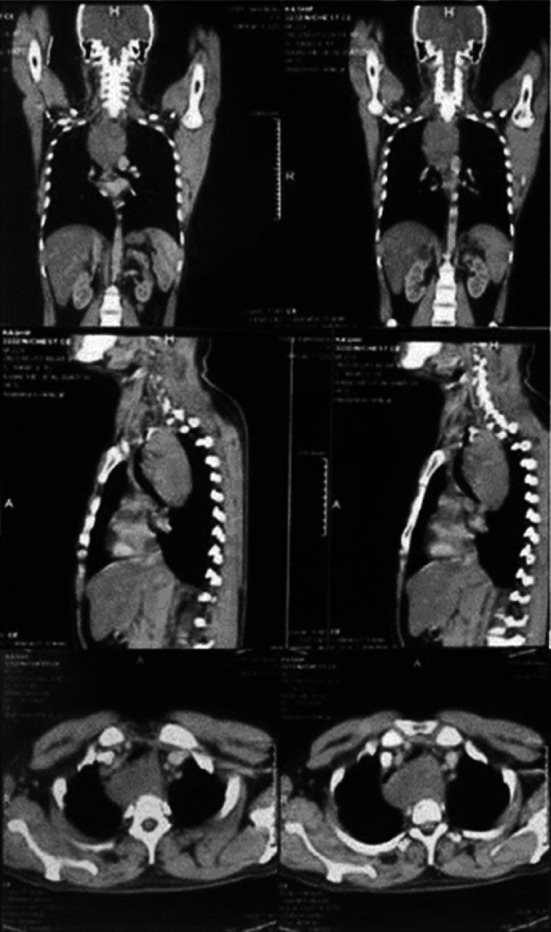
Computed Tomography Scan with contrast.

Upper GI endoscopy revealed a smooth elevated growth with overlying normal mucosa arising from 20 to 30 cm from the incisors, causing luminal compression suggestive of submucosal lesion of esophagus (Fig.1B). Keeping in mind the CT Scan findings, suggestive of leiomyoma based on heterogenicity of the mass and endoscopic findings pointing towards submucosal lesion. Provisional diagnosis of esophageal leiomyoma was made, which is the commonest submucosal tumor of esophagus.

**Fig.1B:**
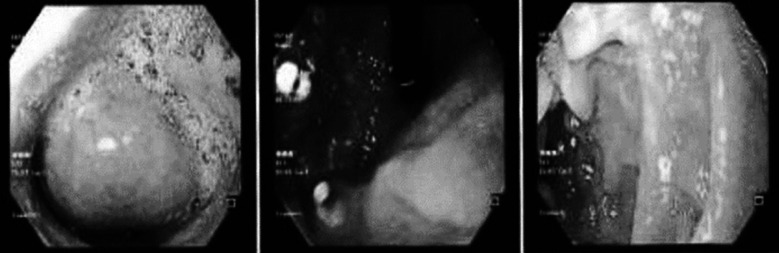
Endoscopy showing a smooth esophageal growth with normal overlying mucosa.

After discussion in the multidisciplinary tumor board meeting, a minimally invasive McKeown esophagectomy was planned. The thoracoscopic phase was undertaken first for tumor dissection. A 11 by 5 cm firm mass was present in the upper thoracic esophagus along with multiple necrotic paraoesophageal lymph nodes, the tumor was adherent to trachea, bilateral bronchi, and aorta. Arch of azygous vein was stretched over the tumor. The lymph nodes were removed with the specimen (Fig.2A) Gastric tube was constructed and esophagogastric anastomosis was performed in the neck after gastric pull-up. The specimen was retrieved via a 5cm transverse abdominal incision.

**Fig.2A:**
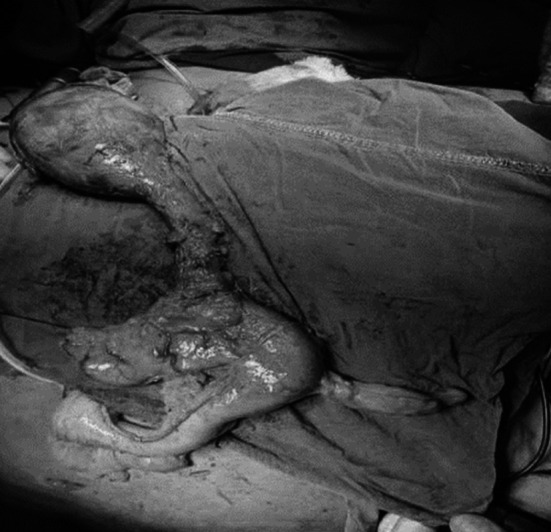
Surgical Specimen: Minimally Invasive Subtotal Esophagectomy.

The postoperative course of the patient was uneventful. He was started on sips on the 4th post-operative day and proceeded to a soft diet on the 6th, which the patient tolerated well and was discharged on the 8th day. Histopathology revealed an encapsulated neoplastic lesion, 11.5 x 7.5 x 5.9 cm in size, composed of spindle-shaped cells arranged in fascicles, nuclear palisading, Verocay body formation, and hyalinized blood vessels. There was no evidence of cytological atypia or malignancy (Fig.2B). The tumor stained positive for SOX10, and negative for CD117 and DOG-1. All these features were consistent with esophageal schwannoma. Moreover, one-year post-procedure the patient remained asymptomatic.

**Fig.2B:**
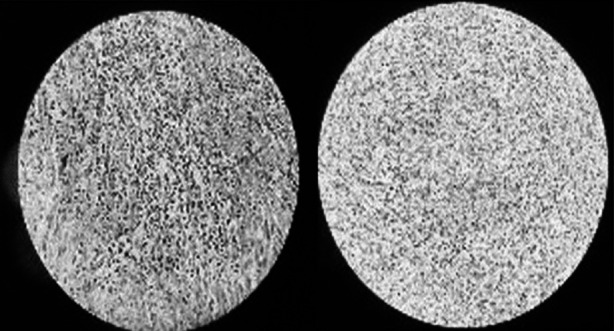
Histopathology: Spindle-shaped cells, arranged in fascicles forming alternating hypocellular &amp; hypercellular areas. SOX10 positive.

## DISCUSSION

In our case, we successfully resected a benign esophageal schwannoma measuring 10.5 cm in its greatest dimension using the McKeown esophagectomy technique. This represents the largest schwannoma removed through a minimally invasive approach, with no intraoperative complications and an uneventful postoperative recovery. This further sets a precedent for managing larger schwannomas using minimally invasive techniques.

Esophageal adenocarcinoma and squamous cell carcinoma account for majority of the esophageal neoplasia. Benign tumors including esophageal schwannomas are rare, accounting for only 2% of all esophageal neoplasms.^1^ Amongst benign tumors, the commonest are leiomyomas accounting for approximately 80%. Choo et al. in a case presentation reported, that there are about 30 reported cases, with most of them originating from Asia.^2^ These tumors arise from Schwann cells of the nerve sheaths and vary in size, with a mean size of 5.6 cm, ranging from 0.5 to 10 cm.^3^ They are typically found in the upper and middle thirds of the esophagus.^4^

Esophageal schwannomas are often asymptomatic due to their small size, but larger tumors may cause symptoms such as dysphagia, dyspnea, cough, weight loss, and chest pain. Although dysphagia is the most commonly reported symptom, dyspnea has also been noted in a few cases as a life-threatening complaint, especially in younger patients.^2^ For instance, Tomono et al. described a case of a 59-year-old woman who was presented with severe airway obstruction requiring immediate intubation which later diagnosed to be due to benign esophageal schwannoma, requiring surgery.^5^ Schwannomas are challenging to diagnose due to their nonspecific characteristics. While imaging modalities such as barium swallow, chest radiography, CT scan, MRI, and endoscopic ultrasound contribute to diagnosis but lack specificity in differentiating schwannomas from other benign tumors. For example, a CT scans may show mass, adherence to adjacent walls, and signs of necrosis or hemorrhage, but these features are not unique to schwannomas. Therefore, a comprehensive diagnostic approach, including clinical evaluation and histopathological confirmation, is necessary.

Moreover, MRI offers detailed images of tumor density and adjacent structures. Esophageal schwannomas may present as hypermetabolic on 18-FDG PET, similar to malignant tumors. Due to the limited accuracy of this technique, endoscopic ultrasound-guided FNA can be used to aid in establishing the pathologic diagnosis. Endoscopic ultrasonography demonstrates the size and the information regarding the origin either of its muscular or submucosal layer. Esophagoscopy usually reveals an esophageal mass with an intact mucosal layer. The diagnosis is confirmed through immunohistochemical studies, where schwannomas typically stain positive for S-100 and SOX-10, unlike CD34, CD117, and desmin, which are markers for leiomyomas.

In our case, the provisional diagnosis based on a CT scan and endoscopic findings was esophageal leiomyoma, given its prevalence as the most common benign tumor. However, due to scope limitations, an endoscopic ultrasound could not be performed, which might have provided further clues regarding the tumor’s origin. Nevertheless, postoperative histopathology and immunohistochemistry confirmed the diagnosis of schwannoma.

The preferred treatment option for the majority of schwannomas is enucleation either endoscopic or surgical.^6^ Endoscopic enucleations is limited only to smaller, well-circumscribed tumors. Surgical modalities include thoracoscopic removal of schwannoma by either Video Assisted Thoracoscopic Surgery (VATS) or Robot-Assisted Thoracoscopic Surgery (RATS), minimally invasive esophagectomy, and open surgical resection. Enucleation performed via a minimally invasive thoracoscopic approach is associated with fewer postoperative complications and shorter hospital stays compared to the open thoracotomy.^7^ However, this too is limited by tumor size and location. For tumors larger than 5 cm in size or involving the upper and middle thirds of the esophagus, tumor excision or esophagectomy are considered better management options.

Thoracotomy, though effective, carries a higher risk of post-operative complications and longer recovery time because of its large-sized incision and a greater circumferential defect in the remaining esophagus. Therefore, total thoracic esophagectomy with cervical esophagogastrostomy could be considered one of the best management options for larger tumors.^8^ This can be performed via an open surgical approach or minimally invasive esophagectomy. Thoracoscopic and laparoscopic techniques are recommended as standard procedures in well-developed and experienced centers. Although rare, there are a few reported cases of malignant esophageal schwannoma.^9^

When an esophageal submucosal tumor is suspected to be malignant based on concerning preclinical or radiographic findings, such as local invasion or enlarged lymph nodes, lymph node dissection should be considered. Radical esophagectomy with regional lymph node dissection may also be needed in certain cases to minimize recurrence. In our patient, lymph node dissection was performed due to the presence of multiple lymph nodes. To date, the largest esophageal schwannoma removed via minimally invasive techniques, as reported by Khan et al. measured 8.0 × 5.5 × 6.5 cm.^10^ Our case now represents the largest esophageal schwannoma successfully removed through minimally invasive McKeown esophagectomy, with no intraoperative complications and a smooth postoperative recovery.

## CONCLUSION

Esophageal schwannoma is a rare entity. A differential diagnosis of esophageal schwannoma should always be considered in benign esophageal tumors, especially in young men. Moreover, in cases where an accurate diagnosis cannot be obtained preoperatively, intraoperative pathologic diagnosis, including the use of a frozen section, should be considered. Surgical procedure choice depends on the size and location of the schwannoma, and minimally invasive esophagectomy is a valid option for larger tumors.

## Data Availability Statement:

Data supporting the study results can be provided followed by a request sent to the corresponding author’s e-mail.

### Author Contributions:

**SQ:** Conceptualization, surgery, writing- review and editing.

**SK:** Data compilation, Literature search, writing- review and editing.

**WAA:** writing- original draft preparation.

**MS:** Literature search, writing- review and editing. All authors have read and approved the final version and SQ is responsible for integrity of the study.
